# Supplementary material for: Programmed cell death-1 is involved with peripheral blood immune cell profiles in patients with hepatitis C virus antiviral therapy
Source: PLoS One. 2024 May 23;19(5):e0299424. doi: 10.1371/journal.pone.0299424 (PMC11115325; doi:10.1371/journal.pone.0299424)
Supplement: S1 Table — (DOCX) [file pone.0299424.s010.docx]

**S1 Table. Patient characteristics for DCV/ASV treatment**

|  | | | All cases  (*n* = 40) | |  |
| --- | --- | --- | --- | --- | --- |
| Age (years) | | | 64.5 (40–79) | |  |
| Sex (male/female) | | | 18/22 | |  |
| Pre-treatment: naïve/IFN+RBV/IFN monotherapy/other  Chronic hepatitis/cirrhosis  Histologically analyzed chronic hepatitis/cirrhosis  Non-histologically analyzed chronic hepatitis/cirrhosis  History of HCC: yes/no  SVR/non-SVR  Mutation: yes (Y93H or L31M)/no | | | 11/20/4/6  23/17  14/3  16/7  1/39  30/10  6/34 | |  |
|  | SVR  (*n* = 30) | non SVR  (*n* = 10) | | *P*-value | |
| Age (years) | 64.5 (40–79) | 66.0 (50–75) | | NS | |
| Sex (male/female) | 15/15 | 3/7 | | NS | |
| Pre-treatment: Naïve/IFN+RBV/IFN/other  Chronic hepatitis/cirrhosis  Liver biopsy: yes/no  History of cancer: yes/no  AST (IU/L)  ALT (IU/L)  Total bilirubin (mg/dL)  Albumin (g/dL)  Platelet count (×10^4^/μL)  Post-treatment:  AST (IU/L)  ALT (IU/L)  Total bilirubin (mg/dL)  Albumin (g/dL)  Platelet count (×10^4^/μL) | 8/15/3/4  19/11  14/16  0/30  59.5 ± 37.4  55.8 ± 31.8  0.8 ± 0.4  3.8 ± 0.5  14.7 ± 5.7  27.7 ± 9.9  22.9 ± 11.8  0.84 ± 0.2  4.26 ± 0.36  15.3 ± 5.8 | 3/5/1/1  4/6  3/7  1/9  42.3 ± 15.9  43.3±21.0  0.9 ± 0.4  4.1 ± 0.3  11.4 ± 3.7  37.1 ± 14.2  42.2 ± 25.1  1.00 ± 0.45  4.27 ± 0.25  11.7 ± 3.69 | | NS  NS  NS  NS  NS  NS  NS  NS  NS  <0.05  <0.01  NS  NS  NS | |

Abbreviations: ALT, alanine aminotransferase; AST, aspartate aminotransferase; HCC, hepatocellular carcinoma; DCV/ASV, daclatasvir and asunaprevir; IFN, interferon; NS, not significant; RBV, ribavirin; SVR, sustained virological response
